# Thiamine as a Possible Neuroprotective Strategy in Neonatal Hypoxic-Ischemic Encephalopathy

**DOI:** 10.3390/antiox11010042

**Published:** 2021-12-25

**Authors:** Gian Pietro Sechi, Flaminia Bardanzellu, Maria Cristina Pintus, Maria Margherita Sechi, Maria Antonietta Marcialis, Vassilios Fanos

**Affiliations:** 1 Department of Medical, Surgical and Experimental Sciences, University of Sassari, 07100 Sassari, Italy; 2 Neonatal Intensive Care Unit, AOU and University of Cagliari, SS 554 km 4500, 09042 Monserrato, Italy; bardanzellu.flaminia@virgilio.it (F.B.); cristina.pintus@yahoo.it (M.C.P.); ma.marcialis@libero.it (M.A.M.); vafanos@tin.it (V.F.); 3 Department of Medical Sciences and Public Health, University of Cagliari, 09124 Cagliari, Italy; margherita.sechi92@gmail.com

**Keywords:** thiamine, neuroprotection, newborn, asphyxia, hypoxic-ischemic encephalopathy

## Abstract

On the basis that similar biochemical and histological sequences of events occur in the brain during thiamine deficiency and hypoxia/ischemia related brain damage, we have planned this review to discuss the possible therapeutic role of thiamine and its derivatives in the management of neonatal hypoxic-ischemic encephalopathy (HIE). Among the many benefits, thiamine *per se* as antioxidant, given intravenously (IV) at high doses, defined as dosage greater than 100 mg IV daily, should counteract the damaging effects of reactive oxygen and nitrogen species in the brain, including the reaction of peroxynitrite with the tyrosine residues of the major enzymes involved in intracellular glucose metabolism, which plays a key pathophysiological role in HIE in neonates. Accordingly, it is conceivable that, in neonatal HIE, the blockade of intracellular progressive oxidative stress and the rescue of mitochondrial function mediated by thiamine and its derivatives can lead to a definite neuroprotective effect. Because therapeutic hypothermia and thiamine may both act on the latent period of HIE damage, a synergistic effect of these therapeutic strategies is likely. Thiamine treatment may be especially important in mild HIE and in areas of the world where there is limited access to expensive hypothermia equipment.

## 1. Introduction

Perinatal asphyxia affects about 4 million newborns worldwide. Among them, in developed nations, between 0.5 and 2/1000 of full-term babies experiencing asphyxia undergo hypoxic-ischemic encephalopathy (HIE), which represents one of the most commonly recognized causes of neonatal death or adverse neurodevelopmental outcome [1,2]. In particular, HIE mortality rate ranges between 10 and 60% and, among surviving neonates, 25% develop neurological sequelae such as infantile cerebral palsy, epilepsy and learning disabilities [2,3].

HIE damage occurs gradually and progressively in distinct phases, acute insult, latent period, secondary and tertiary phase, which evolve over several weeks after acute ischemia [1]. In particular, a detailed analysis of the temporal progression of biochemical processes underlying HIE-related brain damage allowed the introduction in clinical practice of effective therapeutic strategies useful to counteract cell damage and, at the same time, facilitate and enhance cellular protection and repair [4,5]. The main pathophysiological determinants of HIE-related brain damage include oxidative stress, glutamatergic excitotoxicity, cytotoxic/vasogenic edema, cerebral reperfusion/reoxygenation, inflammation, severe mitochondrial dysfunction, and the activation of pro-apoptotic mechanisms until neuronal death [4,5].

Currently, moderate therapeutic hypothermia (TH) is the treatment of choice for perinatal HIE. This treatment mainly works on the latent period of HIE damage and is able to reduce cytotoxic/vasogenic edema, the release of excitatory neurotransmitters and oxygen-related free radicals, the activation of cytokines and globally to improve cerebral metabolism and the overall outcome [6].

However, the available clinical trials indicate that full-term babies affected by mild hypoxic-ischemic encephalopathy show an optimal response to hypothermic treatment, while, the highest percentage of newborns affected by moderate to severe encephalopathy seem not to show high benefits or not respond at all. Therefore, the development of innovative, molecularly targeted, neuroprotective strategies seems necessary and highly promising [7]. Recently, many pharmacologic neuroprotective strategies have been suggested for neonatal HIE, usually to be given in combination with TH (Table 1) [8,9,10,11,12,13,14,15,16,17,18,19,20,21,22,23].

Up till now, the effect of high thiamine doses (vitamin B1), a micronutrient essential for human health and normal brain development. [24,25], on neonatal HIE remains unknown. Indeed, no preclinical or clinical studies are available in literature on the effect of thiamine and its derivatives in neonatal HIE. Importantly, in animals resuscitated from cardiac arrest, a kind of preclinical model of HIE, administration of thiamine improved survival and neurological outcome; moreover, investigations on pathological histology documented less ischemic brain injury, compared with vehicle-treated controls [26] Considering these findings, and on the basis that similar biochemical and histological sequence of events occur in the brain during thiamine deficiency (TD) and hypoxia/ischemia related brain damage, both at synaptic and intracellular level, [27,28,29,30,31], in this review we suggest a probable therapeutic role of high doses parenteral thiamine, defined as dosage greater than 100 mg intravenous daily, in the clinical management of neonatal HIE. Moreover, we will discuss the physiological and biochemical bases of the action of thiamine and its biologically active phosphorylated derivatives in the main phases of this encephalopathy.

## 2. Methods

An electronic literature search was conducted on Medline (via PubMed) and Google Scholar using the cross-referenced combination of the following MeSH terms: thiamine, thiamine deficiency, perinatal asphyxia, perinatal hypoxic-ischemic encephalopathy, term neonates, pathophysiology, treatment. Two review authors (G.P.S. and F.B.) screened and selected publications for inclusion, mainly original articles or previous reviews of the subject, preclinical and clinical studies were included. All authors were involved in data extraction.

Selection of material for inclusion was based on originality, quality, and relevance to the topic. In particular, publications about the terms neonates and neonatal period (birth to 28 days, according to the American Academy of Pediatrics), were carefully assessed.

The searches included studies published between 1980 and October 2021, there were no language restrictions. A total of 120 articles were included, 97 were used as references, of these, 42 covered thiamine function and metabolism, and 55 covered perinatal hypoxic-ischemic encephalopathy pathophysiology and treatment.

## 3. Neonatal Hypoxic-Ischemic Encephalopathy

HIE is caused by intrapartum and neonatal asphyxia, which results in deprivation of blood supply, glucose and oxygen to the brain. The severity of brain damage and neonatal survival are strictly related to the duration and importance of cerebral hypoperfusion [32,33]. In particular, an acute, near-total moderate asphyxial insult leads to cellular damage preferentially at subcortical areas of the brain (e.g., basal ganglia, thalami, mammillary bodies), while a prolonged, partial moderate insult damages preferentially the “so-called” watershed regions of cerebral hemispheres (i.e., areas at the end of artery distribution). Severe, acute or prolonged asphyxial insults, instead, are rare, and lead to extensive cortical/subcortical pathology and frequently result in neonatal death [32,33]. Of note, the incidence of HIE has decreased in recent years [34,35]; however, still relevant differences persist between high- and low-income countries, with higher-income countries showing greater survival [35,36]. Disadvantaged antepartum socioeconomic conditions, such as a high incidence of postpartum thiamine deficiency in specific populations, less access to TH and a global lower quality of the health system in many low-income countries could partly explain this phenomenon [35,36,37]. The pathophysiology of HIE is complex and its knowledge remains incomplete. Experimental models of HIE and clinical studies have led clinicians to divide the temporal sequences of brain injury into four distinct phases [38,39]. In the acute phase/insult, a decreased cerebral blood flow, for more than 10 min (because a cerebral hypoperfusion for less than 10 min usually has no major side effects), significantly reduces the delivery of oxygen and glucose to the brain, with consequent impairment of glucose metabolism in cytoplasma and mitochondria of neuronal and glial cells. In particular, the earliest biochemical change is likely the decrease in α-ketoglutarate-dehydrogenase complex (KGDHC) enzymes activity (tricarboxylic acid/TCA cycle) in mitochondria. This is because KGDHC is the primary site of control of the metabolic flux through the TCA cycle, it is tightly regulated by substrate availability and, among the other major enzymes involved in intracellular glucose metabolism (i.e., pyruvate-dehydrogenase and transketolase), it is more sensitive to antemortem hypoxia [40,41,42]. As a result, a severe impairment of mitochondrial oxidative phosphorylation and, ultimately, of intracellular glucose metabolism occurs, with a dramatic decrease in adenosine triphosphate (ATP) production in cells and a rapid shift towards intracellular anaerobic glucose metabolism, increased lactate production by both neurons and astrocytes, intracellular and extracellular accumulation of lactate, reductions in pH, and focal and systemic acidosis [24,25,43]. Since the energy requirements for the maintenance of ionic gradients across the cell membrane are extremely high, the shortage of ATP leads to sodium and calcium entry into cells and neuronal depolarization and, furthermore, to the impairment of many astrocyte-related functions, such as the control of intracellular and extracellular glutamate concentrations and of the blood-brain barrier (BBB) permeability [24,43]. This complex dysregulation of many cellular functions at different levels triggers a sequence of biological changes leading to cytotoxic edema, glutamate-mediated excitotoxicity, disruption of BBB, vasogenic edema, and oxidative stress with excess production of cellular reactive oxygen species (ROS) and reactive nitrogen species (RNS). Notably, when at a higher degree of cellular oxidative stress, due to the increased production and/or a decreased elimination of ROS and RNS (of particular relevance for understanding the pathophysiology of HIE), there is the generation of peroxynitrite, produced from the reaction of nitric oxide with superoxide [24,43,44]. Indeed, this highly unstable compound is able to progressively inactivate the major enzymes involved in intracellular glucose metabolism, including KGDHC, via nitration of the tyrosine residues of the enzymes at physiological pH [44]. Importantly, nitrotyrosine, a reaction product of peroxynitrite and proteins, has been found in the brain tissue of full-term neonates after perinatal asphyxia [45], and infants with HIE who do not show initial recovery of cerebral oxidative metabolism have extremely poor outcomes [46]. 

In most neonates, after an acute phase of injury, an adequate cerebral blood flow resumes again with the delivery of oxygen and glucose to the brain, and a partial recovery of the impaired metabolism with resolution of the acute cell swelling, over approximately 30–60 min [38,39]. However, the metabolization of the compounds produced during the acute phase of injury, and of glucose and oxygen remains inadequate, likely due to the residual damage or insufficiency/shortage of major enzymes and coenzymes, such as KGDHC and thiamine pyrophosphate (TPP), involved in intracellular glucose metabolism. In neonates with perinatal asphyxia, this abnormal metabolic state could account for the phenomenon in which an excessive oxygen supplementation, following a period of oxygen deficiency, may augment the cerebral injury [46,47,48]. Moreover, it can activate further specific, dangerous or protective molecular pathways, in a following short “latent” phase, typically lasting approximately from 1 h to 6 h. During this phase many brain cells show partial recovery of oxidative metabolism; although, with persistent residual mitochondrial injury and the occurrence of an apparent cerebral inflammation with increased microglial reactivity and production of pro-inflammatory cytokines. Moreover, in this phase, EEG activity remains suppressed, high-energy phosphates are near normal and there is activation of both adenosine A1 receptors, which mediate the initial suppression of neural activity and it is, in turn, an important protective mechanism, and of multiple programmed cell death pathways [46,49,50]. 

Importantly, in HIE in the frame time of the initial 6 h, the therapeutic use of moderate cerebral hypothermia initiated as early as possible, and continued for a sufficient duration to allow a significant reduction in cerebral metabolism, in parallel with reduction of the excito-oxidative cascade, is able to induce a potent, long-lasting neuroprotection [46,49]. The therapeutic effect and safety of this treatment, has been validated by several large multicenter trials [51,52,53] and the evidence of reduced brain injury on modern neuroimaging [54,55]. 

Furthermore, these studies document that the effectiveness of TH is strictly related to the promptness of its application, and that the earlier it is used the better the long-term outcome [49,51]. This is because the metabolic imbalance underlying the pathophysiology of HIE appear to be progressive and, in newborns with moderate to severe injury, the “latent” phase is frequently followed by a secondary deterioration many hours later lasting from approximately 6 h to 15 h [49,51]. This secondary energy failure is characterized by near-complete failure of oxidative metabolism in mitochondria and a progressively severe oxidative stress, accumulation of glutamic acid in brain interstitial fluid, formation of new cytotoxic and vasogenic edema, appearance of stereotypic seizures and eventual spreading of programmed cell death [49,51]. In particular, among the multiple biochemical mechanisms involved, of particular relevance seem to be the near-complete inactivation of the major enzymes involved in intracellular glucose metabolism, due to the reaction of peroxynitrite with the tyrosine residues of the enzymes, the excessive sequestration of calcium by mitochondria and the permeabilization of mitochondrial membranes (the “intrinsic” apoptosis pathway), in parallel with the activation of cell surface death receptors triggered by the concomitant inflammation (the “extrinsic apoptosis pathway) [49,56]. Of note, several experimental and clinical data indicate that in HIE during the time frame of the secondary energy failure, in many crucial specific brain areas, there is a definite transition from the so-called stage of “reversible biochemical lesions” to the stage of structural, irreversible lesions, with a high probability of permanent neurological sequelae or a fatal outcome [49,51]. 

After the secondary energy failure, and the probable occurrence of irreversible lesions in critical brain areas, a tertiary/chronic phase occurs, during the following months after the acute insult, and involves further cell death and astrogliosis with remodeling of the injured brain [39]. 

## 4. Thiamine

Humans cannot synthesize thiamine and are reliant on exogenous sources. Tissue storage of this vitamin is very limited, thus depletion of the body stores can occur rapidly after 2–3 weeks of unbalanced nutrition in healthy individuals, and after 3–5 days in patients with chronic diseases [24,25]. The half-life of thiamine after intravenous or oral administration is short, 96 min and 154 min, respectively [57].

Thiamine, and its phosphorylated intracellular derivatives, thiamine monophosphate (TMP), TPP (also named thiamine diphosphate), and triphosphate (TTP) are water-soluble organic molecules essential for normal cellular functions, growth and development in all tissues [58]. In the human body these crucial molecules can act in a myriad of biological settings and may have either a coenzyme or a non-coenzyme action [59]. In particular, at physiological pH, free thiamine has a positive charge and a definite antioxidant and anti-inflammatory activity strictly dose related. Notably, the antioxidant activity of free thiamine seems more effective for RNS [60]. Moreover, this vitamin may affect interneuronal transmission, by its role in the generation of some neurotransmitters (e.g., acetylcholine and serotonin), and neural membranes functions, because thiamine is a structural component of mitochondria and synaptosomes. Moreover, it may foster hippocampal neurogenesis [24,61,62].

**Figure 1:**
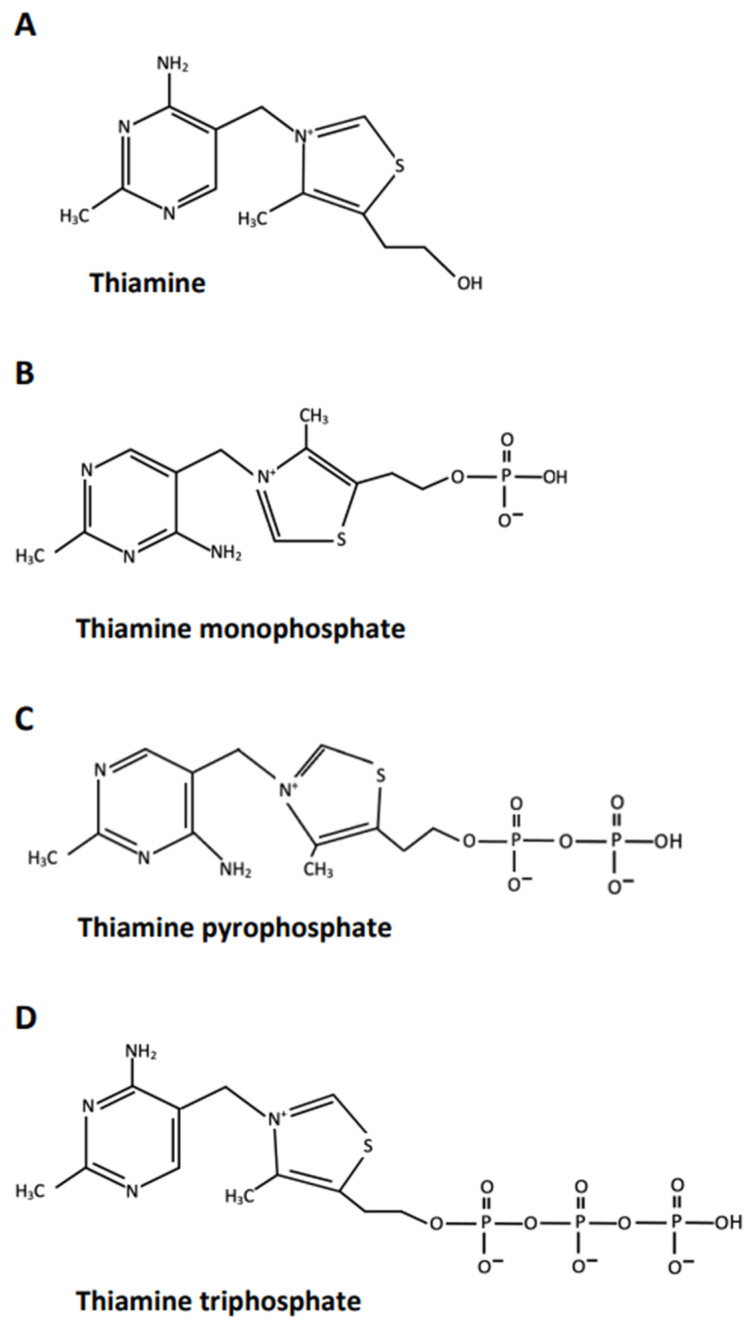
Chemical formula and main biological functions of thiamine and its phosphorylated derivatives. (**A**) Thiamine: dose related antioxidant and anti-inflammatory activity; a role in interneuronaltransmission, neural membranes function, and hippocampal neurogenesis. It is not a coenzyme; it has a positive charge. (**B**) Thiamine monophosphate: essential for transfer of thiamine across cellular membranes. It is not a coenzyme; it is a neutral molecule. (**C**) Thiamine pyrophosphate (or diphosphate): It functions as a coenzyme in several biochemical pathways in the brain, essential for synthesis of nucleotides, glutathione, lipids/myelin, ATP for oxidative energy production, several amino acids and some neurotransmitters. TPP also has a direct antioxidant activity *per se*. It has a negative charge. (**D**) Thiamine triphosphate: may activate high-conductance chloride channels; may have a role in cell energy metabolism. It is not a coenzyme, it has two more negative charges.

TMP instead is a neutral molecule essential for transfer of thiamine across cellular membranes [58]. TPP is synthesized in the cytosol and is transported by specific carriers in mitochondria. It has a negative charge and is the most abundant form of thiamine in the body (>80%). TPP is the main biologically active form of thiamine and, in concert with magnesium, functions as an essential coenzyme in several biochemical pathways in the brain, in particular, in the metabolism of carbohydrates, lipids, and amino acids. Specifically, in carbohydrate metabolism, TPP acts both at cytoplasmic level, in glycolysis and pentose phosphate pathways for nucleotides, glutathione and lipids/myelin synthesis, and at mitochondrial level, in oxidative phosphorylation and TCA cycle for synthesis of ATP and oxidative energy production, synthesis of amino acids and glucose-derived neurotransmitters, such as glutamic acid and GABA [25]. Of note, the tripeptide glutathione, produced by TPP in the pentose phosphate pathway has powerful antioxidant activity against both cellular ROS and RNS [63]. Additionally, TPP is able to exert a direct antioxidant effect *per se*, as discussed below. In mitochondria, TPP may be further phosphorylated to TTP, which has two more negative charges and thus may activate high-conductance anion/chloride channels in astrocytes and neurons and play a role in regulating cholinergic and serotonergic neurotransmission. Moreover, TTP may have a role in cell energy metabolism, via its back-transformation in TPP (Figure 1) [58].

Thiamine requirement is related to the total caloric intake and the proportion of calories provided as carbohydrates [24]. Thus, high caloric and high carbohydrate diets increase the demand for thiamine. The recommended dose of thiamine for an average, healthy adult is 1.4 mg per day. This dose is higher in children, in critically ill conditions, in individuals with excess metabolic demand, and during pregnancy and lactation [24]. The clinical settings in which a definite TD can occur are myriad. These include a poor dietary intake, chronic alcohol misuse with malnutrition, poor gastrointestinal absorption (e.g., recurrent vomiting, chronic diarrhea, gastro-intestinal surgical procedures), increased utilization (e.g., thyrotoxicosis, chronic systemic infections, pregnancy), cancer and chemotherapeutic treatments, magnesium depletion, use of specific chemical compounds and drugs (e.g., some diuretics, tolazamide), chronic dialysis, THTR2 gene mutations [24,64,65].

Definite TD can cause serious, treatable diseases such as Wernicke’s encephalopathy (WE), peripheral neuropathy, and specific cardiovascular disturbances. In particular, severe, short-term TD commonly induces WE, whereas a mild to moderate, prolonged deficiency preferentially leads to damage to peripheral nerves [24,25]. WE is an acute life-threatening disorder fully responsive to prompt and adequate thiamine replacement. Untreated WE has an estimated mortality of 20%, and about 80% of patients who survive develop Korsakoff’s syndrome (KS), the chronic irreversible form of WE that does not remit with thiamine replacement. Of note, some cases of WE inappropriately treated with low doses of thiamine may also develop KS [24]. Importantly, the diagnosis of WE, and thiamine treatment should mandatorily be made within the first two weeks after the onset of initial symptoms/signs indicative of possible TD, during the time frame so-called “stage of reversible biochemical lesions”, when high-doses parenteral thiamine can completely reverse brain damage. Whereas, after about two weeks, irreversible structural brain lesions in specific diencephalic and brainstem areas usually occur, with consequent lack of therapeutic efficacy of thiamine replacement [24,25,66].

## 5. Hypoxia-Ischemia and Thiamine Deficiency in the Brain: Similar Biochemical and Histological Lesions

Past neuropathological investigations in animals and humans dating back to the 90s [27,28], and recent neuroimaging findings [67,68,69], indicate that the morphological changes in selective brain areas, such as the basal ganglia, mammillary bodies, medial thalami and the brainstem olivary complex due to TD and those due to hypoxia-ischemia may be identical. In particular, this kind of focal, selective damage in subcortical areas of the brain is a well-known distinctive pattern of WE and it is commonly observed in the majority of newborns with perinatal asphyxia when the hypoxic-ischemic insult occurs acutely. Whereas, when a subacute or chronic hypoxia-ischemia happens, which occurs in about 10% to 15% of newborns with perinatal asphyxia, this leads to preferential damage at the borders of the vascular beds of cerebral arteries. [32,70,71]. In both disorders, at the cellular level, the earliest biochemical change is likely the decrease in KGDHC enzymes activity in mitochondria of astrocytes in individuals with TD [24], and in mitochondria of astrocytes and neurons in neonates with perinatal hypoxia-ischemia [42] with consequent impairment of mitochondrial oxidative phosphorylation and, ultimately, of intracellular glucose metabolism [25]. Of note, a decrease in the activity of the other two major enzymes involved in cellular glucose metabolism, the pyruvate-dehydrogenase complex and transketolase, is noticed later after the decrease in the activity of KGDHC [24,25,42]. 

As a result, this acute metabolic derangement produces a global decrease in the use of glucose in the brain [24,72], with consequent severe impairment of energy metabolism in astrocytes and neurons both in HIE and in TD, with progressive activation of an excito-oxidative cascade, due to mitochondrial impairment, the occurrence of secondary subacute inflammation and eventually apoptotic cell death [24,49,73]. 

In both disorders, useful biomarkers of mitochondrial energy failure and a decrease in ATP production are the plasmatic increase of lactate and pyruvate levels, which in daily clinical practice indicates the opportunity to use high doses of parenteral thiamine as potential beneficial treatment.

Together, these findings are congruent with recent observations that TD *per se* is able to modulate the activity of the Hypoxia Inducible Factor-1α (HIF-1α) [22,29], as likewise it occurs after brain hypoxia-ischemia in full-term human neonates [74,75]. HIF-1α is the main transcription factor involved in hypoxic stress, which regulates the expression of genes involved in pro-inflammatory, pro-apoptotic and pro-survival responses [76]. The precise mechanisms underlying HIF-1α mediated neuroprotection or injury remain unclear and they may be partly related to the maturation stage of the brain at the time of the hypoxic-ischemic insult [74]. Most in vitro and in vivo findings suggest that the stabilization and activation of specific HIF-1α signaling may play an important role in promoting neuronal survival in both HIE and thiamine deficiency either by up-regulation of protective or repair genes, such as vascular-endothelial growth factor and erythropoietin, or by reduced expression of pro-apoptotic HIF-1α target genes and hence apoptotic cell death [22,29,31,74]. Of note, in TD in particular, thiamine replenishment in astrocytes is able to inhibit this specific pro-apoptotic mechanism [22,29].

## 6. Thiamine and Its Derivatives in the Management of Neonatal HIE

In recent years, the documented beneficial effect of TH in moderate HIE has led clinicians and researchers to evaluate and discuss cooling in combination with other pharmacologic neuroprotective agents in order to enable further improvements in outcome for infants with HIE [73,77]. However, among these compounds, thiamine has still not been considered [36,49,73,77], despite the well definite neuroprotective properties of this vitamin [25,58]. The findings extensively discussed in this review strongly suggest that thiamine given at high dosages should be included among the potential neuroprotective agents for infants with HIE, either as a complementary or an alternative treatment to TH.

This micronutrient is essential, at low concentrations, for several physiological cellular functions and development in all human tissues, in particular the brain and hearth, because of the intense metabolic demand of the nervous and cardiovascular systems [25,58]. Instead, experimental and clinical evidence indicates that very high dosages of thiamine are needed to reverse and treat the biochemical cellular lesions that may happen in pathological conditions, such as the occurrence of subacute encephalopathy/Wernicke’s encephalopathy and cardiomyopathy due to severe subacute TD [24,25]. 

**Figure 2:**
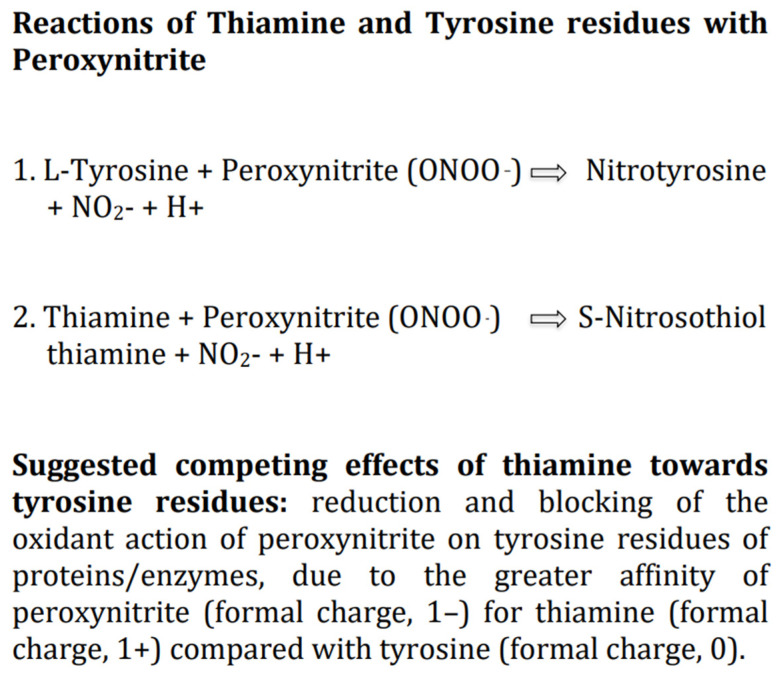
Competing effects of thiamine on tyrosine residues peroxynitrite binding. For reactions 1 and 2, see also references [60,80].

Progressive oxidative stress in mitochondria and the cytoplasm of astrocytes and neurons, leading to excessive formation of ROS and RNS, including the very dangerous compound peroxynitrite, is likely the main pathophysiologic determinant of brain damage in HIE [38,45,73]. We suggest that thiamine *per se* as antioxidant, given intravenous at high doses, should counteract potently the damaging effects of ROS and RNS, including the reaction of peroxynitrite with the tyrosine residues of the major enzymes involved in cellular glucose metabolism, which is the likely earliest biochemical change in HIE [24,42,58,59]. This is supported by several experimental studies on diverse oxidants which document that thiamine at high dosages is able to diminish or counteract the production of both ROS and RNS, although it is much more effective toward RNS [60,78]. Of note, among thiamine derivatives, TPP is also able to exert both a direct antioxidant effect *per se* and an indirect antioxidant activity, acting as an essential coenzyme, in the pentose phosphate pathway, for the synthesis of a major cellular antioxidant, the tripeptide glutathione [25]. Thus, in newborns with HIE, to boost in the brain the antioxidant defense mechanisms by increasing the levels of thiamine and TPP should limit the amount of ROS/RNS to a level that is not threatening for the integrity of lipids, proteins, carbohydrates and nucleic acids [79]. Additionally, among the myriad of possible molecular interactions between thiamine and biological compounds, of particular relevance for treatment of HIE is the well documented interaction between thiamine and peroxynitrite [60]. Indeed, thiamine may compete with the tyrosine residues of the major enzymes involved in cellular glucose metabolism to bind peroxynitrite anions (ONOO^–^) and activate different oxido-reductive biochemical pathways less dangerous for cell viability [60,80]. In particular, it is hypothesizable a greater affinity, at physiological pH. of the negatively charged peroxynitrite (formal charge, 1–) for the positively charged free thiamine (formal charge, 1+) compared with tyrosine which is a neutral molecule (formal charge, 0). (Figure 2) Studies on TD provide indirect support for an interaction of peroxynitrite with thiamine [60]. In particular, the finding that in neurons within susceptible brain areas deficiency of thiamine induces tyrosine nitration [81].

In addition to the well-defined role in counteracting oxidative stress, high doses of thiamine and its derivatives may have other multiple potential benefits in infants with HIE, which include fostering the activity of the major enzymes involved in intracellular glucose metabolism, a definite anti-inflammatory activity by differential regulation of pro-inflammatory mediators, a modulatory effect on interneuronal transmission, the enhancement of hippocampal neurogenesis and the maintenance of mitochondrial function by recovery of mitochondrial network architecture, membrane potential and respiration [58,59,61,82,83], thus avoiding potential mitochondrial failure, and in turn preventing neuronal and glial cell death [84].

## 7. Thiamine Neonatal Requirements

In the neonatal period (birth to 28 days) TD is rare, as usually thiamine levels are higher in newborns and in cord blood due to the preferential delivery of this vitamin to the fetus at the expense of the mother [85,86,87,88]. This biological mechanism, which in humans is the preferential distribution of thiamine to the developing tissues, highlights the importance of optimizing the intake and status of this vitamin in the mother and the newborn in order to foster, in this crucial period for acquisition of fundamental cognitive skills, an optimal neurodevelopmental outcome [89,90,91]. However, this mechanism does not preclude in the newborn the occurrence of TD when the mother, and her breast milk, are deficient in this vitamin and the infant is exclusively breastfed, as may frequently happen in low-and middle-income countries [87]. Other clinical settings that may predispose to TD in this age group, also in high-income countries, are total parenteral nutrition or enteral nutrition, genetic inborn errors of thiamine metabolism, gastrointestinal disorders and acute critical illness, including pediatric emergency room, surgical settings, pediatric cardiology and nephrology/dialysis [88].

The suggested adequate intake of thiamine for pregnant women and healthy infants showing ≤ 6 months of age is 1.4 mg/d and 0.2 mg/d, respectively [92]. However, the exact requirement of thiamine in a newborn with HIE, who develops a severely altered hypermetabolic state, is unknown, but should surely be much greater than 0.2 mg/d [24,49]. Of note, in infants with HIE low serum magnesium levels have also been documented [93]. This specific deficiency may lead to an impaired response to thiamine until magnesium is given [24]. 

Considering that the body’s stores of thiamine in humans are only sufficient for up to 18 days [24] after birth, in a full-term infant with deficient intake of this vitamin, it is conceivable that the earliest clinical manifestations of TD usually present at about one month of age [87,88]. Early clinical signs may be refusal to breastfed, irritability, vomiting and persistent crying that is difficult to console. [87,88]. Importantly, the progression of clinical symptoms/signs to overt infantile beriberi is a medical emergency, as infants can die within hours of clinical presentation if not promptly treated [88]. The clinical presentation is broad and may include cardiovascular disturbances, such as critical heart failure, shock and pulmonary arterial hypertension, aphonia, absent deep-tendon reflexes, nystagmus, ophthalmoplegia, altered consciousness, seizures, respiratory symptoms and hyperlactatemia with acidosis [87,88]. High doses of parenteral thiamine supplementation promptly reverses these clinical disturbances and the hyperlactatemia [24,87,88]. 

Therapeutic Doses of Thiamine in Neonates with HIE

The optimum dose of thiamine in neonates with HIE is unknown. The route of administration should be parenteral, preferentially by infusion over a period of at least 30 min., with thiamine hydrochloride dissolved in normal saline, three times per day for at least 72 h—the standard time of TH. Parenteral administration allows a rapid and regular entry of thiamine in the brain mainly by passive diffusion [24]. Considering that high thiamine doses are needed to reverse and treat the biochemical cellular lesions that may happen in pathological conditions [24], there are no known adverse effects related to intake of high doses of this vitamin [94] and that very high doses of thiamine (until 300 mg per day) are routinely used and regarded as safe in daily clinical practice in infants with infantile beriberi, aged 0–6 months [95,96,97], a plausible, safe dose in neonates with HIE should be 50 mg of intravenous thiamine three times per day, for 72 h, when used as a complementary treatment to TH, or for 5 days when used as an alternative treatment to TH [35,36,95,96,97].

Moreover, given the essential role of magnesium in intracellular synthesis of TPP from thiamine, in all instances, serum levels of magnesium should be checked, and supplemented when necessary [24]. 

## 8. Conclusions

**Figure 3:**
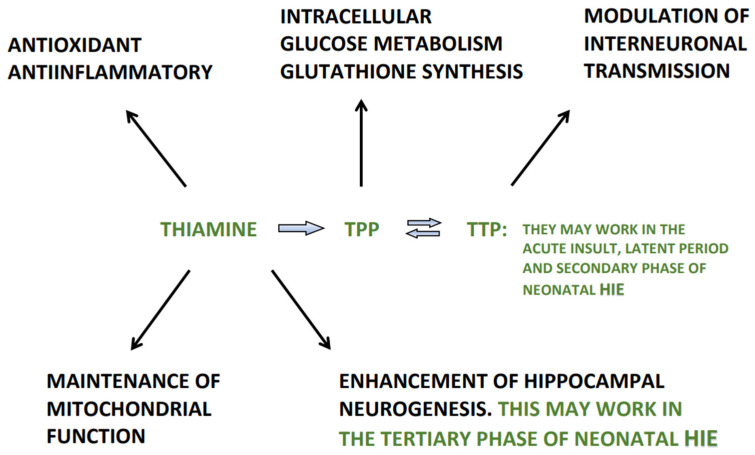
Main mechanisms of action of thiamine and its derivatives in the different phases of neonatal HIE. TPP = thiamine pyrophosphate; TTP = thiamine triphosphate.

Experimental studies and the available clinical trials indicate that TH alone fails to provide an effective neuroprotection in about half of the neonates with HIE, thus new compounds that could work in synergy with TH able to provide additive neuroprotection are needed. On the basis that similar biochemical and histological sequence of events occur in the brain during TD and hypoxia/ischemia related brain damage, in this review we discuss a probable therapeutic role of thiamine and its derivatives in the clinical management of neonatal HIE. We suggest that thiamine *per se* as an antioxidant, given intravenously at high doses, should potently counteract the toxic effects of ROS and RNS in the brain, including the reaction of peroxynitrite with the tyrosine residues of the major enzymes involved in cellular glucose metabolism, which plays a key pathophysiological role in HIE in neonates. Other potential benefits in infants with HIE, related to thiamine and its derivatives, include fostering the activity of the major enzymes involved in intracellular glucose metabolism, a definite anti-inflammatory activity, a modulatory effect on interneuronal transmission, the enhancement of hippocampal neurogenesis and the maintenance of mitochondrial function, (Figure 3) thus avoiding a potential mitochondrial failure, and in turn preventing neuronal and glial cell death.

Notably, thiamine may work on several phases of neonatal HIE damage, including the acute insult, the latent period and the secondary phase. Given that thiamine and TH may both act on the latent period of HIE damage, a synergistic effect of these therapeutic strategies on HIE is likely. Importantly, due to its low cost and ease of delivery, the use of high thiamine doses, as complementary and/or alternative treatment to TH, may be especially important in mild HIE and in areas of the world where there is limited access to expensive hypothermia equipment and services. Future studies evaluating the possible therapeutic effect of high thiamine doses in neonatal HIE are warranted in animal models and human pilot studies. Given the established safety profile of thiamine, which is routinely used in neonates in many clinical settings, promising preclinical studies could quickly lead to human clinical trials to evaluate this B vitamin in neonatal HIE, in the first instance as an adjunct treatment to therapeutic hypothermia.

## Figures and Tables

**Table 1:** Types of Neuroprotective Agents Suggested for Neonatal HIE.

Agents	Type of Study	Main Mechanisms of Action	References
Xenon/Argon	Prec./Clin.	NMDA-receptor antagonists; anti-inflammatory; improve apoptosis	Amer et al. (2018) [8] Broad et al. (2016) [9]
Magnesium	Preclinical	NMDA-receptor antagonist; anti-oxidant; anti-inflammatory	Koning et al. (2019) [10]
Allopurinol	Preclinical	Antioxidant; possible gender effect	Rodriguez-Fanjul et al. (2017) [11]
Cannabinoids	Preclinical	Modulation excitotoxicity, oxidative stress, inflammation	Pazos et al. (2013) [12]
Erythropoietin	Preclinical	Antiapoptotic, antioxidant, anti-inflammatory; neurovascular remodelling; promotes neural stem cell proliferation	Xiong et al. (2019) [13]
Melatonin	Preclinical	Antioxidant, anti-inflammatory.	Robertson et al. (2020) [14]
Topiramate	Clinical	Anticonvulsant; anti-excitotoxicity	Filippi et al. (2020) [15]
Phenobarbital	Preclinical	Anticonvulsant; reduced cerebral metabolic demand; antioxidant	Barks et al. (2010) [16]
N-acetylcysteine	Preclinical	Antioxidant, anti-inflammatory; restores intracellular glutathione	Wang et al. (2007) [17]
Indometacin	Preclinical	Anticaspase activity and antiapoptotic; restores intracellular glutathione	Tetorou et al. (2021) [18]
Pentoxifylline	Preclinical	Phosphodiesterase inhibitor; anti-inflammatory	Tetorou et al. (2021) [18]
Quercetin/Coumestrol	Preclinical	Flavonoid/isoflavonoid antioxidants, anti-inflammatory	Tetorou et al. (2021) [18]
Polyphenols	Preclinical	Antioxidant, anti-inflammatory, antiapoptotic properties	West et al. (2007) [19]
2-Iminobiotin	Preclinical	Inhibition of cytochrome C-caspase 3 neuronal death pathway in female. Gender specificity	Nijboer et al. (2007) [20]
NTR5221/NCT01626934	Preclinical	Antioxidants by inhibition of nitric oxide synthesis	Faviè et al. (2018) [21]
GSK360A	Preclinical	Brain-targeted hypoxia-inducible factor1α-stabilization by inhibition of prolyl-4-hydroxylase	Kuan et al. (2021) [22]
Stem Cells	Preclinical	Modulation of immune/inflammatory response,	Nair et al. (2021) [23]
